# MOMENTARY INTERPERSONAL EMOTION REGULATION IN AN ADULT LIFESPAN SAMPLE

**DOI:** 10.1093/geroni/igac059.499

**Published:** 2022-12-20

**Authors:** Claire Growney, Tammy English

**Affiliations:** Washington University in St. Louis, St. Louis, Missouri, United States; Washington University in St. Louis, St. Louis, Missouri, United States

## Abstract

Older adults are theorized to maintain emotional well-being by drawing on their available resources, such as social partners who support their emotional goals. In the present study, adults (N = 290) age 25-85 completed an experience sampling procedure (6x/day for 10 days) in which they reported their current emotions and use of interpersonal emotion regulation (IER) strategies. Unexpectedly, older age was associated with lower overall use of IER strategies. Results from multilevel models indicate that within-person associations between IER strategy use and positive affect were strongest among older adults, with older adults experiencing highest positive affect on occasions where they report engaging in IER. Between-person associations between IER strategy use and negative affect were only present among younger and middle-aged adults, indicating that younger individuals who use IER more on average tend to have higher negative affect. Findings highlight the role of social partners in older adults’ emotional wellbeing.

